# Participatory budgeting with cumulative votes

**DOI:** 10.1007/s11238-025-10097-1

**Published:** 2025-10-31

**Authors:** Piotr Skowron, Arkadii Slinko, Stanisław Szufa, Nimrod Talmon

**Affiliations:** 1 University of Warsaw, Warsaw, Poland; 2 University of Auckland, Auckland, New Zealand; 3 Jagiellonian University, Kraków, Poland; 4 Ben-Gurion University, Beersheba, Israel

**Keywords:** Single Transferable Vote, Participatory Budgeting, Additive Utilities

## Abstract

In participatory budgeting a group of decision makers is given a set of projects—each with a cost—and has to decide which projects should be funded within the constraints of an available budget. Each decision maker, in some form, expresses their preference over the projects. The goal of an aggregation procedure is to select—based on voter preferences—a subset of projects whose total cost does not exceed the budget and optimal in some sense. We propose several aggregation methods based on the idea of cumulative votes, e.g., for the setting when each of the *n* voters is given one coin equal to $(\nicefrac {1}{n})$th of the budget and she specifies how this coin should be split among the projects. We compare our aggregation methods based on (1) axiomatic properties, and (2) computer simulations. We identify one class of methods, Minimal Transfers, that demonstrates particularly desirable behaviour. In particular, it satisfies a strong notion of proportionality, and, thus, is promising for its use in practice.

## Introduction

The idea of participatory budgeting (PB) originated in Brazil in the 1980s, when political reformers sought ways to move beyond the legacy of the military dictatorship (1964–1985), which had been marked by exclusion and corruption (Wampler, 2012). Their aim was to make local decision-making more transparent and to strengthen social justice and democracy (Cabannes, 2004). In this context, PB emerged as a natural innovation.

In the initial stage of PB, governments and civil society organizations define a set of goals based on certain principles, without yet referring to the budget. For instance, many PB programs in Brazil rely on the Quality of Life Index, first developed by the local government in Belo Horizonte. These goals are then translated into specific projects to be put forward for public selection. In some European cities, for example, in Poland, citizens themselves can also propose projects (Walczak & Rutkowska, 2017). The municipality subsequently reviews these proposals, filtering out inadmissible ones and assigning costs to those that are approved. Several researchers (e.g., Sintomer et al. (2012)) emphasise importance of deliberation but this rarely happens. The second stage is actual voting in which citizens express their views on the relative importance of the projects on offer. Thus, Participatory Budgeting is indeed a direct-democracy approach to budgeting.

The most prominent applications of PB have been at the municipal level, where residents collectively decide how to allocate a fraction of the city’s budget. Beyond this setting, PB-inspired methods have also been applied in diverse contexts, such as: (1) an airline company choosing which movies to include in its in-flight entertainment system (Skowron et al., 2016), where licensing costs vary across films; (2) a fan-owned soccer club deciding which athletes to sign; (3) generating a concise collection of statements that proportionally reflects societal viewpoints (Fish et al., 2024; Boehmer et al., 2025); and (4) selecting validators in proof-of-stake blockchain protocols (Cevallos & Stewart, 2020; Burdges et al., 2020).

Recently, PB has attracted considerable attention, and an increasing share of public funds is now allocated through this process.^1^ This growing interest is also reflected in the scientific literature. Most research has focused on procedures based on approval ballots, where each voter selects a subset of projects they consider worth funding (sometimes subject to the constraint that the chosen projects do not exceed the available budget or that a certain number of projects needs to be approved). To a lesser extent, scholars have examined ordinal ballots, where voters rank (a subset of) projects from most to least desirable (Aziz & Shah, 2020; Rey & Maly, 2023). Several works have examined the use of cardinal utilities, where voters assign numerical scores to projects (Fain et al., 2018; Fluschnik et al., 2019; Benade et al., 2017; Peters et al., 2021).

In this paper, we study a model in which each of the *n* voters is endowed with virtual coins worth *L*/*n*, where *L* denotes the budget limit. Each voter distributes these coins across the projects, thereby indicating the intensity of their preferences. By analogy with voting theory (see, e.g., (Cole Jr, 1949)), we refer to such ballots as cumulative votes. Cumulative votes are closely related to cardinal ballots, with the key distinction that cardinal ballots are not required to sum to a fixed predefined value. This difference is significant, as it influences both the formulation of axioms and the design of voting rules. Finally, we note that even when voters cast approval ballots, it is often reasonable to interpret or convert them into cardinal or cumulative ones. For example, Faliszewski et al. (2023) argue that larger and more expensive projects tend to be more valuable from a voter’s perspective, and therefore propose interpreting a voter’s utility for a project as proportional to its cost.

We begin our study of aggregation methods for PB with cumulative votes by examining three greedy methods, analogous to top-*k* multiwinner voting rules (Faliszewski et al., 2017). We refer to these rules as (1) greedy by support, (2) greedy by excess, and (3) greedy by support over cost.

These methods are intuitive, easy to explain, and computationally efficient. However, they also have important shortcomings. In particular, as we will show, they fail to satisfy several desirable axiomatic properties and can, in some cases, completely ignore the preferences of minority groups.

To address this limitation, we propose several adaptations of the Single Transferable Vote (STV) rule to the PB setting. STV is a well-known ordinal-based multiwinner election rule, widely used in contexts where proportional representation is desired, such as parliamentary elections. Our adaptations differ in several key details; here we informally describe one of them. Given a budget limit *L*, each of the *n* voters is endowed with a bag of *L*/*n* coins, which they can distribute among the available projects as they wish. We then consider the coin stash accumulated by each project. In each iteration, if there exists a project whose stash meets or exceeds its cost, that project is funded, and any surplus coins are redistributed to other projects according to the preferences of the voters who contributed to it. If no project can be funded, we instead eliminate the project with the smallest stash, redistribute its coins to other projects, and continue iteratively.

Since cumulative votes generalize both approval and ordinal ballots,^2^ our methods apply to these models as well. In practice, this means that voters need not use the full expressive power of cumulative ballots: they may simply provide approval or ordinal preferences, which can then be translated, by the user interface or by the aggregation algorithm, into cumulative form. At the same time, voters who wish to express their preferences in a more detailed manner retain the option to do so.

We illustrate the behavior of our rules by analyzing several desirable properties that a PB procedure should satisfy.

1. **Resistance to to agenda manipulation**. A recurring concern in PB is the filtering of projects by the steering committee or project proposers (Kurdys-Kujawska et al., 2019). While such filtering may be necessary if the number of submitted projects is too large, it also creates opportunities for agenda manipulation. For instance, the committee may merge multiple projects into one or split a large project into several smaller ones. Hence, robustness of the aggregation procedure to such merging and splitting is an important property.
2. **Monotonicity**. Another desirable property is responsiveness to changes in voters’ preferences, commonly referred to as monotonicity. If a voter increases their support for a given project, this should not decrease that project’s chances of being funded.
3. **Proportionality**. Finally, fairness to minorities is crucial, typically formalized as proportionality. This property requires that any sufficiently large minority of voters, if coordinated, can ensure that projects they strongly support will be funded.

We identify one class of rules, Minimal Transfers, which behaves particularly well with respect to our axiomatic properties and which indeed produces proportional results.

## Formal model

In our model there is a set of projects $P = \{p_1, \ldots , p_m\}$; the cost of a project $p \in P$ is a natural number, denoted *c*(*p*). There is a set of *n* voters $V = \{v_1, \ldots , v_n\}$, where voter $v_j$ expresses her preferences over the projects by assigning a value $v_j(p)$ to each $p \in P$ such that $v_j(p) \ge 0$ and $\sum _{p \in P} v_j(p) = 1$ (notice that we refer to both the *j*th voter and her cumulative vote using the same symbol $v_j$); intuitively, the value of $v_j(p)$ is understood as the fraction of the funds in disposal of voter $v_j$ that the voter would like to assign to project *p*.^3^ We say that a voter $v_i$ *supports* a project *p* if $v_i(p) > 0$. The above notation naturally extends to sets. For each $B \subseteq P$ and each $v_j \in V$ we denote $v_j(B) = \sum _{p \in B}v_j(p)$ and $c(B) = \sum _{p \in B} c(p)$.

A *budgeting scenario* is a tuple $E=(P, V, c, L)$, where *P*, *V*, and *c* are as defined above, and $L \in \mathbb {N}$ is a *budget limit*. An *aggregation method* $\mathcal {R}$ is a function that, given a budgeting scenario, selects a *bundle* of projects $\mathcal {R}(E) \subseteq P$ such that $c(\mathcal {R}(E)) \le L$.

## Desired properties

We compare our methods against several axiomatic properties, focusing on three main categories:

1. axioms that guard against agenda manipulation,
2. axioms related to monotonicity, and
3. axioms concerning proportionality.

The first category addresses potential manipulation by the steering committee when setting the agenda. Monotonicity axioms capture the principle that collective outcomes should respond positively to increases in individual support. Proportionality axioms ensure fairness toward minority groups. Both monotonicity and proportionality are widely recognized as highly desirable in the context of participatory budgeting. Since our focus is on cumulative votes, we adapt these axioms to this setting in the following section.

### Resistance to agenda manipulation axioms

The following axiom guards, to some extent, against agenda manipulation by the steering committee, which might otherwise attempt to block a project from being funded by splitting it into multiple subprojects.

#### Definition 1

(Resistance to Splitting) An aggregation method $\mathcal {R}$ satisfies *resistance to splitting* if for each budgeting scenario $E = (P, V, c, L)$, each funded project $p \in \mathcal {R}(E)$, and for each budgeting scenario $E'$ which is obtained by splitting project *p* into a set of projects $P'$ whose combined cost is $c(P')=c(p)$, and for each voter $v_i$ its combined support from voter $v_i$ is $v_i(P') = v_i(p)$, it holds that $\mathcal {R}(E') \cap P' \ne \emptyset$.

This axiom is closely related to splitting monotonicity introduced by Rey et al. (2020) for approval ballots. In contrast to the approval setting, our axiom must specify how votes cast on a project *p* are translated into votes on the set of projects $P'$ obtained by splitting *p*. We impose only a weak requirement: namely, for every voter *i*, the total weight of their votes for $P'$ must equal their vote for *p*, i.e., $v_i(P') = v_i(p)$. This condition does not prescribe how the votes are distributed among the individual projects in $P'$. By comparison, in the approval-ballot version of the axiom, a voter who approves *p* is assumed to approve each project in $P'$.

Our next axiom helps guard against agenda manipulation by the steering committee, which might attempt to block a project from being funded by merging it with another one.

#### Definition 2

(Resistance to merging) An aggregation method $\mathcal {R}$ satisfies *resistance to merging* if for each budgeting scenario $E = (P, V, c, L)$, each $P' \subseteq \mathcal {R}(E)$, and each scenario $E' = (P \setminus P' \cup \{p'\}, V, c', L)$ such that $p'$ is a new project which costs $c(P')$ and such that for each voter $v_i$ we have that $v_i(p') = v_i(P')$, it holds that $p' \in \mathcal {R}(E')$.

This axiom very closely relate to merging monotonicity by Rey et al. (2020) for approval ballots with the difference in how the votes for a merged project $p'$ are constructed form the votes cast on individual projects from $P'$.

### Monotonicity axiom

Roughly speaking, monotonicity requires that increasing support for a funded project should not reduce its chances of being funded. Formally, consider a budgeting scenario $E = (P, V, c, L)$. We say that a voter $v \in V$ increases their support for a project $p \in P$ if, instead of casting ballot *v*, they submit a ballot $v'$ such that: (1) $v'(p) > v(p)$, and (2) $v'(p') \le v(p')$ for all $p' \in P \setminus {p}$.

#### Definition 3

(Support monotonicity) An aggregation method $\mathcal {R}$ satisfies *support monotonicity* if for each budgeting scenario $E = (P, V, c, L)$, each project $p \in \mathcal {R}(E)$, and each budgeting scenario $E' = (P, V', c, L)$ where in $V'$ voter *v* increases her support for *p*, ceteris paribus, then $p \in \mathcal {R}(E')$.

This axiom adapts classical notions of monotonicity from the social choice literature. It is most closely related to candidate monotonicity studied in the context of multiwinner elections with ranked preferences (Elkind et al., 2017). The key difference is that in candidate monotonicity, increasing support for one candidate only affects the position of a single other candidate—the one that is overtaken in some voter’s ranking. By contrast, in our setting, increasing support for a project *p* may come at the expense of reducing support for multiple other projects simultaneously.

### Proportionality axioms

Next, we introduce three proportionality axioms, which differ in the degree of coordination required among a group of voters for them to secure control over a certain fraction of the budget.

#### Definition 4

(Weak Proportional Representation) An aggregation method $\mathcal {R}$ satisfies *Weak Proportional Representation (Weak-PR)* if for each budgeting scenario $E = (P, V, c, L)$, each subset of voters $V' \subseteq V$, and each set of projects $P' \subseteq P$ with $c(P') \le L \cdot \nicefrac {|V'|}{n}$, there exist a scenario $E'$ which differs from *E* only in the votes of the voters from $V'$, such that $P' \subseteq \mathcal {R}(E')$.

#### Definition 5

(Proportional Representation) An aggregation method $$\mathcal {R}$$ satisfies *Proportional Representation (PR)* if for each budgeting scenario $$E = (V, P, c, L)$$, each subset of voters $$V' \subseteq V$$, and each set of projects $$P' \subseteq P$$ with $$c(P') \le L \cdot \nicefrac {|V'|}{n}$$, and such that all voters $$v' \in V'$$ support all projects in $$P'$$, and no other projects (that is, for each $$v' \in V'$$ it holds that $$v'(p') >0 $$ if and only if $$p' \in P'$$) we have that $$P' \subseteq \mathcal {R}(E)$$.

Our strongest proportionality axiom is presented below. There, we relax the requirement on the total cost of the projects in $P'$. According to Strong-PR, groups of voters do not need to coordinate in detail to secure projects they favor; intuitively, it suffices that they agree on the set of projects to which they assign positive support.

#### Definition 6

(Strong Proportional Representation) An aggregation method $$\mathcal {R}$$ satisfies *Strong Proportional Representation (Strong-PR)* if for each scenario $$E = (P, V, c, L)$$, each subset of voters $$V' \subseteq V$$ and each subset of projects $$P' \subseteq P$$ such that all voters $$v' \in V'$$ support all projects in $$P'$$, and no other projects (that is, for each $$v' \in V'$$ it holds that $$v'(p') >0 $$ if and only if $$p' \in P'$$) it holds that either $$P' \subseteq \mathcal {R}(E)$$ or for each $$p \in P' \setminus \mathcal {R}(E)$$ we have that $$c(p) + c(P' \cap \mathcal {R}(E)) > L \cdot \nicefrac {|V'|}{n}$$.

It is straightforward to observe that any aggregation rule satisfying Strong-PR also satisfies PR, and any rule satisfying PR in turn satisfies Weak-PR.

Similar axioms have been studied in the context of approval-based PB rules (Brill et al., 2023). The closest related notion in the literature is extended justified representation (EJR) for cardinal ballots (Peters et al., 2021). EJR is logically incomparable with our axioms. On the one hand, it is weaker, since it requires voters to coordinate on the exact values they assign to particular projects. On the other hand, it is stronger, as it allows conclusions to be drawn about groups of projects that are not exclusively supported by the voters. Moreover, EJR is formulated in terms of the overall utility enjoyed by a minority group, whereas our axioms provide explicit guarantees about which projects will be selected from given sets.

## The rules

In this section, we define and discuss our aggregation methods for PB with cumulative votes.

### Greedy rules

In this section, we take what is arguably the most straightforward approach: adapting well-known greedy algorithms for participatory budgeting (Goel et al., 2019) to the setting of cumulative ballots. We begin by introducing the general class of greedy rules, each defined by a specific function that determines the priority of projects.

Let *f* be a function that assigns to each project *p* a real number, called the *priority* of *p*. A greedy rule based on *f* first orders the projects in descending order of their priorities. It then iterates through this ranked list,^4^ deciding in each step whether the currently considered project will be selected. Formally, let *L*(*t*) denote the remaining budget at the beginning of the *t*-th iteration (with $L(1) = L$). Suppose the rule examines project *p* in iteration *t*. If $c(p) \le L(t)$, then *p* is selected and the remaining budget is updated to $L(t+1) = L(t) - c(p)$. Otherwise, *p* is not selected, the budget remains unchanged, $L(t+1) = L(t)$, and we move to the next project in the priority-ranked list.

In what follows, we define three aggregation methods, each corresponding to a greedy rule with a different choice of priority function.

- **Greedy-by-support (GS).** This is the greedy rule based on $f_{\textrm{GS}}(p) = \sum _{j \in [n]} v_j(p)\cdot (L/n)$. This rule prioritise the projects the highest total support from the voters.
- **Greedy-by-support-over-cost (GSC).** It is based on $f_{\textrm{GSC}}(p) = \nicefrac {1}{c(p)} \cdot \sum _{j \in [n]} (v_j(p) \cdot (L / n))$. This rule prioritise the projects with the highest fraction of their cost funded by the voters.
- **Greedy-by-excess (GE).** It is based on the priority function $f_{\textrm{GE}}(p) = \sum _{j \in [n]} (v_j(p) \cdot (L / n)) - c(p)$. This rule prioritise the projects for which the excess of the total funds contributed to it over the costs is largest.

#### Remark 1

We do not consider Greedy-by-Excess-over-Cost as it is equivalent to GSC.

The first rule (GS) can be viewed as an adaptation of Knapsack Voting (Goel et al., 2019) to the setting of cumulative ballots. However, all three rules share an important drawback: a substantial minority of voters may be effectively ignored if their support is spread too thinly across many projects. We illustrate this issue with the following example.

#### Example 1

Consider a set *P* of 20 projects, all having the same cost equal to one, and a set of 100 voters with the following preferences: The first 60 voters consider the first 10 projects excellent and they all decide to assign the value $\nicefrac {1}{10}$ to each of them. The remaining 40 voters have quite opposite preferences—they decide to put the utility of $\nicefrac {1}{10}$ on each of the last 10 projects. The budget limit is $L = 10$. Here, GS, GSC, and GE would select the first 10 projects for funding, thus effectively ignoring the opinion of a large fraction of the society.

Example 1 can also be used to demonstrate the lack of proportionality in other rules proposed in the literature. One such method, often regarded as proportional, is based on maximizing the *smoothed Nash welfare (SNW)* (Fain et al., 2018; Fluschnik et al., 2019) using cardinal utilities. If we interpret cumulative ballots directly as cardinal utilities, the rule operates as follows. For each budgeting scenario (*P*, *V*, *c*, *L*), SNW returns a bundle *B* of projects whose total cost does not exceed *L* and which maximizes the product

$$\begin{aligned} \prod _{v_j \in V} \Big (v^*_j + \sum _{p \in B} v_j(p)\Big ), \end{aligned}$$

where $v^*_j$ is the maximum value that voter *j* can obtain in any feasible outcome. When applied to the scenario from Example 1, SNW would disproportionately favor the majority: it would select eight projects supported by 60% of the voters and only two projects supported by the remaining 40%. Thus, although somewhat less severely, SNW still exhibits the same negative feature as the greedy rules.

In the next section, we address the shortcomings of the greedy rules GS, GSC, and GE by proposing alternative aggregation methods inspired by the Single Transferable Vote (STV). These methods are designed to mitigate the disproportionality observed in Example 1. In particular, in that instance, our rules would allocate the budget to six projects supported by the 60% of voters and to four projects supported by the remaining 40%, thereby achieving a more proportional outcome.

### Cumulative single transferable vote (CSTV) rules

In this section, we present an adaptation of the Single Transferable Vote (STV) rule to participatory budgeting with cumulative votes. We refer to this adaptation as *Cumulative-STV* (CSTV). We begin by outlining the general scheme of the rule and then discuss several variants that differ in key aspects of the algorithm.

All variants of CSTV are built on the following principle: each of the *n* voters should be able to decide on the allocation of a $\nicefrac {1}{n}$-fraction of the total budget. Accordingly, we say that a project *p* is *eligible for funding* if

$$\begin{aligned} \textrm{support}(p) = L \cdot \frac{\sum _{j=1}^n v_j(p)}{n} \ge c(p) \text {.} \end{aligned}$$

Observe that the total cost of all projects eligible for funding never exceeds the budget. At first glance, one might consider the simple rule of selecting exactly those projects that are eligible and rejecting all others. However, this naive strategy often produces undesirable outcomes. For instance, suppose there is a large set of high-quality projects, each supported by nearly all voters, and that voters distribute their support roughly uniformly among them. In such a case, it may happen that no single project meets the eligibility condition, leading the rule to return an empty set of funded projects. This would happen, for instance, in Example 1.

To address this and related issues, our algorithm allows for certain transfers of cumulative ballots among projects. Some transfers are more straightforward and unobjectionable than others. The guiding idea behind these permissible transfers is as follows. Since voters lack coordination mechanisms, they may collectively allocate excessive funds to certain projects. If a voter knew that her contribution to a project was unnecessary—because the project would be funded without it, or because it would not be funded even with it—she would likely prefer to redirect her support toward other projects she values. The CSTV rules account for such oversupply of funds and perform these redistributions automatically on behalf of the voters.

We now describe the generic CSTV rule. To instantiate it as a concrete rule, one must specify the following components (or, from an algorithmic perspective, subroutines):

1. the project-to-fund selection procedure,
2. the excess-redistribution procedure,
3. the no-eligible-project procedure, and
4. the inclusive-maximality postprocedure.

While the excess-redistribution procedure is always the same for all variants of our rules, the other subroutines have multiple variants. We discuss these subroutines in detail in the remainder of this section.

The generic scheme proceeds as follows. Initialize $S = \emptyset$. Then repeat the following steps until a stopping condition is met.

1. **If there exists an eligible project.** Select one such project *p* according to the *project-to-fund selection procedure*.
  - If the total support for *p* strictly exceeds its cost (i.e., $\textrm{support}(p) > c(p)$), then for each voter $v_j$ with $v_j(p) > 0$, transfer part of their support from *p* to other projects that $v_j$ initially supported, so as to bring $\textrm{support}(p)$ as close to *c*(*p*) as possible. These transfers are carried out according to the *excess-redistribution procedure*.
  - Add *p* to *S*, remove it from further consideration, reduce the remaining budget by setting $L:= L - c(p)$, and charge the voters for *p*, i.e., for all $v_j$, set $v_j(p) = 0$.
2. **If no project is eligible for funding.** Perform the *no-eligible-project procedure*, which consists of one of the following actions:
  - eliminate the least popular project *p* and transfer the values assigned to *p* by the voters to their other supported projects, or
  - select the most popular project *p* and transfer values from other projects to *p* so that it becomes eligible for funding.

After executing the chosen action, return to the beginning of the loop. Finally, once a stopping condition is reached, it may still be the case that the remaining budget suffices to fund additional projects. In such instances, the resulting bundle of selected projects is not *inclusive maximal*. To address this, one may either invoke the *inclusive-maximality postprocedure* or allow the unused portion of the budget to be carried over (e.g., to the following year’s budget).

Below, we describe the individual components of the generic CSTV rule introduced above.

#### Project-to-fund selection procedure

If multiple projects are eligible for funding, we select the one with the highest priority, as determined by one of the following three functions: (1) $f_{\textrm{GS}}$, (2) $f_{\textrm{GSC}}$, or (3) $f_{\textrm{GE}}$, which were introduced in Sect. 4.1 in the context of greedy rules.

#### Excess redistribution procedure

We employ the *proportional strategy* for redistributing excess support. Formally, let *p* be the project currently selected for funding, and let

$$\begin{aligned} \textrm{tran}(p) = \{v_j \mid v_j(p)> 0 \ \text {and} \ \exists p' \notin S: v_j(p') > 0 \} \end{aligned}$$

denote the set of voters who allocate part of their support to *p* as well as to at least one other not yet selected project.

On behalf of the voters, the rule redistributes the excess by scaling down contributions to *p* proportionally to voters’ initial support. Specifically, we determine $\gamma < 1$ such that

$$\begin{aligned} \frac{\gamma L}{n} \sum _{v_j \in \textrm{tran}(p)} v_j(p) \;+\; \frac{L}{n} \sum _{v_j \notin \textrm{tran}(p)} v_j(p) \;=\; c(p). \end{aligned}$$

Intuitively, $\gamma$ is the scaling factor that ensures *p* receives support exactly equal to its cost. For each $v_j \in \textrm{tran}(p)$, the surplus $(1-\gamma )\cdot v_j(p)$ is then redistributed among all not-yet-selected projects, proportionally to the initial support that $v_j$ assigned to them.

Other redistribution strategies are also natural. For example, one may consider an additive version of proportional shares, which we term the *equal-shares strategy*. The idea is to make voters pay for the selected project in shares as equal as possible. Formally, we find $\lambda$ such that

$$\begin{aligned} \frac{L}{n} \sum _{v_j \in \textrm{tran}(p)} \min (v_j(p), \lambda ) \;+\; \frac{L}{n} \sum _{v_j \notin \textrm{tran}(p)} v_j(p) \;=\; c(p), \end{aligned}$$

and, for each $v_j \in \textrm{tran}(p)$ with $v_j(p) > \lambda$, we redistribute the surplus $(v_j(p) - \lambda )$ among all not-yet-selected projects, proportionally to the initial support that voter $v_j$ assigned to them.

Another possibility would be to adopt an egalitarian criterion, aiming to minimize the maximum transfer required from any voter. While we do not pursue these alternative strategies in this paper, we consider their investigation a promising direction for future work.

#### No-eligible-project procedure

We consider two alternative procedures that can be applied when no project is currently eligible for funding. The chosen procedure is executed until some project becomes eligible.

- **Elimination-with-transfers (EwT).** In this procedure, we eliminate a project *p* with either the minimal $\textrm{excess}(p) = \textrm{support}(p) - c(p)$ or the minimal ratio $\textrm{excess}(p)/c(p)$. If the project-to-fund selection procedure is based on the priority function $f_{GE}$, we use the former; if it is based on $f_{GSC}$, we use the latter. Once *p* is chosen for elimination, for each voter $v_j$ who allocated part of their support to *p*, we redistribute this support proportionally among the other projects initially supported by $v_j$. If $v_j$ supported only *p*, then no transfers are made. Note that if, at any point, there exists a project whose cost exceeds the remaining budget, this project will eventually be eliminated and its support redistributed.
- **Minimal-transfers (MT).** We say that a project *p* is *eligible for funding by transfers* if
  $$\begin{aligned} \frac{L}{n} \cdot \sum _{j: v_j(p) > 0} \sum _{\ell =1}^m v_j(p_\ell ) \;\;\ge \;\; c(p). \end{aligned}$$
  Thus, *p* is eligible for funding by transfers if its cost could be covered provided all voters who initially assigned positive support to *p* redirected their remaining support exclusively to *p*. If the project-to-fund selection procedure is based on $f_{GE}$, we select among such projects the one that requires the smallest total amount of transfers to become eligible. If it is based on $f_{GSC}$, we select instead the project *p* with the highest ratio $\textrm{support}(p)/c(p)$. If no project is eligible for funding by transfers, this subroutine stops. Otherwise, we select project *p* as described and perform the redistribution according to the proportional strategy (cf. Sect. 4.2.2). To compute the transfers, we iteratively proceed as follows. Let $r = \textrm{support}(p)/c(p) < 1$. Each supporter $v_j$ of *p* first computes the desired support to assign to *p*:
  $$\begin{aligned} v_j(p) :=\; \min \!\left( \sum _{\ell =1}^m v_j(p_\ell ), \, \frac{v_j(p)}{r}\right) . \end{aligned}$$
  The voter then proportionally transfers support from the other projects they supported to *p*. This process is repeated until $r = 1$.

#### Inclusive maximality postprocedure

We introduce two procedures that can be applied when the algorithm stops, and a part of the available budget remains unused. This situation may arise when voters concentrate their support too narrowly on specific projects (see Example 2, below).

- **Reverse eliminations (RE).** This procedure is applicable only when the Elimination-with-Transfers (EwT) method was previously used for projects that were not initially eligible for funding. We iterate over the non-selected projects in the reverse order of their elimination. Intuitively, projects eliminated earlier received the least support. For each project, we check whether its cost does not exceed the remaining budget, and if so, we fund it. This approach is consistent with the logic of EwT, which can be viewed as implicitly producing a ranking of the projects: whenever EwT funds a project *p*, it places *p* at the topmost available position in the ranking; whenever it eliminates *p*, it places *p* at the bottommost position. Thus, EwT combined with reverse eliminations can be seen as a greedy procedure that continues along this ranking, starting from the first non-selected project.
- **Acceptance of undersupported projects (AUP).** This procedure is applicable only when Minimal-Transfers (MT) was used as the no-eligible-project subroutine. It proceeds analogously to MT, but without requiring the condition of eligibility by transfers. Specifically:

1. If the project-to-fund selection procedure is based on $f_{GE}$, then among all not-yet-selected projects whose costs do not exceed the remaining budget, we select the project *p* that maximizes
  $$\begin{aligned} \frac{L}{n} \cdot \left( \sum _{j: v_j(p) > 0} \sum _{\ell =1}^m v_j(p_\ell )\right) - c(p). \end{aligned}$$
2. If the procedure is based on $f_{GSC}$, then we select the project *p* that maximizes
  $$\begin{aligned} \frac{\frac{L}{n} \cdot \sum _{j: v_j(p) > 0} \sum _{\ell =1}^m v_j(p_\ell )}{c(p)} . \end{aligned}$$

For each voter $v_j$ with $v_j(p) > 0$, we then transfer all of their remaining support from other projects to *p*. The project *p* is added to *S*, and the procedure is repeated until no further project can be added.

We illustrate this step with the following example.

##### Example 2

Suppose there are five projects $p_1, p_2, p_3, p_4, p_5$ with costs 1.5, 1.4, 1.3, 1.2, 1.1, respectively, and five voters $v_1, v_2, v_3, v_4, v_5$. The budget limit is 5, and each voter $v_i$ allocates their share of the budget, equal to 1, entirely to project $p_i$. In this case, the Elimination-with-Transfers procedure does not fund any project. However, if Reverse Eliminations is applied, it will select projects $p_2, p_3, p_4, p_5$ for funding.

### Selection of variants

The various design choices described above give rise to a number of aggregation methods, out of which we consider the following concrete CSTV aggregation methods:

$$\begin{aligned} \textrm{EwT}= & \textrm{GE} + \textrm{EwT} + \textrm{RE},\\ \textrm{EwTS}= & \textrm{GS} + \textrm{EwT} + \textrm{RE},\\ \textrm{EwTC}= & \textrm{GSC} + \textrm{EwT} + \textrm{RE},\\ \textrm{MT}= & \textrm{GE} + \textrm{MT} + \textrm{AUP},\\ \textrm{MTS}= & \textrm{GS} + \textrm{MT} + \textrm{AUP},\\ \textrm{MTC}= & \textrm{GSC} + \textrm{MT} + \textrm{AUP}; \end{aligned}$$

This means, for example, that EwT employs the following subroutines: Greedy-by-Excess (GE) as the project-to-fund selection procedure, Elimination-with-Transfers (EwT) as the no-eligible-project procedure, and Reverse Eliminations (RE) as the inclusive-maximality postprocedure. In addition, we also consider the greedy rules, GS and GSC.

## Analysis of axiomatic properties of the rules

Our analysis is summarized in Table 1, with detailed proofs provided in the appendix. For most properties, our rules do not offer guarantees, and the corresponding proofs consist of small counterexamples. In contrast, the proofs showing that our rules satisfy specific proportionality axioms rely on an invariant: a designated group of voters consistently directs a fixed amount of money toward a corresponding group of projects, and this allocation is never redirected outside of this group.

None of the rules satisfies Support Monotonicity; this is not surprising, given how demanding monotonicity requirements are in voting procedures; indeed, even STV is not monotonic. Further, none of the proportional rules satisfy Resistance to Merging, and only MT satisfies Resistance to Splitting. We believe that weaker variants of these axioms merit further investigation. For example, one could study axioms that impose stricter conditions on how support is redistributed when a project is split, or on how support from other projects can be redirected toward a given one. It is possible that some impossibility theorems are at play here, though they remain to be discovered. As expected, the greedy rules perform poorly with respect to proportionality, whereas the CSTV variants satisfy Weak-PR and PR, with some (such as MT, MTS and MTC) even meeting Strong-PR.

**Table 1:** Axiomatic properties of GS, GSC, and the CSTV variants

	GS	GSC	EwT	EwTS	EwTC	MT	MTS	MTC
Resistance to splitting	✕	$\checkmark$	✕	✕	✕	$\checkmark$	✕	✕
Resistance to merging	$\checkmark$	✕	✕	✕	✕	✕	✕	✕
Support monotonicity	✕	✕	✕	✕	✕	✕	✕	✕
Weak-PR	✕	$\checkmark$	$\checkmark$	$\checkmark$	$\checkmark$	$\checkmark$	$\checkmark$	$\checkmark$
PR	✕	✕	$\checkmark$	$\checkmark$	$\checkmark$	$\checkmark$	$\checkmark$	$\checkmark$
Strong-PR	✕	✕	✕	✕	✕	$\checkmark$	$\checkmark$	$\checkmark$

## Experimental evaluation

**Table 2:** The statistics computed for our rules on data from PabuLib for elections with the number of projects greater than 30

Rule	VS	ER	AC	Cost-Sat	EJR+
Actual	45.5%	19.9%	1.0	1.0	2.845
Equal Shares	45.2%	13.7%	0.537	0.847	0
BOS Equal Shares	46.1%	12.7%	0.601	0.867	0.032
Cost-Utilities					
GS	41.6%	25.2%	1.856	0.942	11.452
GSC	48.1%	14.3%	0.709	0.909	0.333
EwT	40.4%	15.2%	0.444	0.692	0
EwTS	44.9%	20.0%	1.411	0.963	5.224
EwTC	46.2%	13.6%	0.588	0.857	0.059
MT	33.7%	16.5%	0.407	0.532	0.078
MTS	45.9%	13.5%	0.686	0.886	0
MTC	45.2%	12.4%	0.54	0.811	0
Score-Utilities					
EwT	57.5%	15.7%	0.415	0.582	0.032
EwTS	56.3%	20.0%	1.411	0.963	5.224
EwTC	60.8%	14.6%	0.653	0.93	0.037
MT	55.8%	16.5%	0.406	0.526	0.114
MTS	58.1%	13.0%	0.584	0.848	0
MTC	60.8%	12.3%	0.478	0.719	0.009

**Table 3:** The statistics computed for our rules on data from PabuLib for elections with the number of projects lower than 15

Rule	VS	ER	AC	Cost-Sat	EJR+
Actual	65.0%	20.6%	1.0	1.0	0.216
Equal Shares	59.4%	22.1%	0.795	0.825	0
BOS Equal Shares	64.3%	19.2%	0.895	0.931	0.086
Cost-Utilities					
GS	64.0%	21.9%	1.221	1.006	0.555
GSC	62.6%	20.5%	0.843	0.877	0.03
EwT	55.5%	24.2%	0.711	0.719	0
EwTS	67.2%	20.7%	0.854	0.893	0.009
EwTC	60.6%	20.8%	0.799	0.84	0.002
MT	54.6%	24.5%	0.702	0.699	0
MTS	60.3%	21.1%	0.837	0.865	0
MTC	59.7%	21.1%	0.787	0.826	0
Score-Utilities					
EwT	63.1%	24.5%	0.701	0.702	0
EwTS	56.3%	20.0%	1.411	0.963	5.224
EwTC	66.3%	21.7%	0.755	0.792	0
MT	62.8%	24.8%	0.698	0.691	0
MTS	66.8%	21.0%	0.815	0.853	0
MTC	66.5%	21.4%	0.761	0.797	0

**Table 4:** The statistics computed for our rules on data from PabuLib for elections with the number of projects varying between 15 and 30

Rule	VS	ER	AC	Cost-Sat	EJR+
Actual	54.3%	18.0%	1.0	1.0	0.764
Equal Shares	53.2%	14.0%	0.654	0.818	0
BOS Equal Shares	54.9%	12.9%	0.712	0.873	0.008
Cost-Utilities					
GS	50.7%	23.6%	1.682	0.99	2.707
GSC	55.9%	13.5%	0.739	0.873	0.008
EwT	48.6%	15.6%	0.577	0.68	0
EwTS	54.1%	17.8%	1.192	0.966	0.831
EwTC	54.1%	13.7%	0.673	0.828	0
MT	45.3%	16.4%	0.553	0.606	0
MTS	53.9%	13.8%	0.76	0.87	0
MTC	53.9%	13.1%	0.667	0.823	0
Score-Utilities					
EwT	62.5%	16.0%	0.559	0.631	0
EwTS	63.8%	14.1%	0.758	0.907	0.004
EwTC	65.7%	13.6%	0.605	0.751	0
MT	61.5%	16.5%	0.551	0.598	0
MTS	65.0%	13.4%	0.697	0.856	0
MTC	65.9%	13.2%	0.615	0.764	0

To complement the axiomatic analysis provided in the section above, in this section we compare our eight aggregation methods through computer-based simulations. In particular, we report on simulations done on 1398 real-life PB elections, which come from the PabuLib library (Faliszewski et al., 2023).

In PabuLib, each voter *v* assigns to each project *p* a numeric score $\text {sc}_v(p)$. For approval ballots, this value is 1 if *v* approves of *p*, and 0 otherwise. We convert these instances to the cumulative form using one of two approaches, following Faliszewski et al. (2023) and Papasotiropoulos et al. (2025):

- **Cost-utilities.** The value that a voter *v* assigns to project *p* is proportional to its cost:
  $$\begin{aligned} v(p) = \frac{\text {sc}_v(p) c(p)}{\sum _{p' \in P} \text {sc}_v(p') c(p')} \text {.} \end{aligned}$$
- **Score-utilities.** The value that a voter *v* assigns to project *p* is proportional to its score:
  $$\begin{aligned} v(p) = \frac{\text {sc}_v(p)}{\sum _{p' \in P} \text {sc}_v(p')} \text {.} \end{aligned}$$

We consider the following statistics (all values are averages over instances). Given a winning bundle of projects *B*, we compute:

- **Voter satisfaction (VS):** This metric is defined as the average fraction of each voter’s support that goes to funded projects:
  $$\begin{aligned} \frac{1}{n} \cdot \sum _{v \in V}\sum _{p \in B} v(p) \text {.} \end{aligned}$$
- **Exclusion ratio (ER)** (Faliszewski et al., 2023): This metric is defined as the fraction of voters who are completely excluded (i.e., none of their supported projects is funded):
  $$\begin{aligned} \frac{1}{n} \cdot \left| \left\{ v \in V : \sum _{p \in B} v(p) = 0\right\} \right| . \end{aligned}$$
- **Average cost (AC):** This metric is defined as the average cost of a funded project:
  $$\begin{aligned} \frac{1}{|B|} \cdot \sum _{p \in B} c(p) \text {.} \end{aligned}$$
- **Average cost-satisfaction (Cost-Sat)** (Faliszewski et al., 2023): This metric is defined as the average total cost of funded projects supported by a voter. For approval ballots, this corresponds to the average amount of money spent on projects approved by a voter; for cardinal ballots, the values are scaled accordingly:
  $$\begin{aligned} \frac{1}{n} \cdot \sum _{v \in V} \sum _{p \in B} \text {sc}_v(p), c(p) \text {.} \end{aligned}$$
  Since this value is not directly comparable across elections, we normalize it by dividing by the value of the same metric as computed by the greedy rule used in the actual election.^5^ This approach follows Papasotiropoulos et al. (2025).
- **Extended justified representation plus violations (EJR+)** (Brill & Peters, 2023): This metric measures the violations of one of the strongest proportionality axioms studied in the literature. Informally, the axiom requires that if there exists a sufficiently large and cohesive group of voters (i.e., all approving the same projects), then this group must be entitled to decide over a proportionate share of the budget. We use the up-to-one variant of EJR+ adapted to cost-utilities (Brill & Peters, 2023).

We do not include metrics related to resilience against merging or splitting projects, nor to support monotonicity, as it is unclear how to compute such metrics efficiently. Investigating this remains an interesting open problem.

In Table 2, we report the data from elections with more than 30 projects; in Table 3, we provide the corresponding data for elections with fewer than 15 projects; and in Table 4, we present the data for elections with between 15 and 30 projects. This division allows us to compare how our rules behave separately in small, medium, and large elections. We additionally include in our comparison two known rules for participatory budgeting, the ADD1U variant of the method of equal shares (Peters & Skowron, 2020; Peters et al., 2021; Faliszewski et al., 2023), and the recently introduced method of equal shares with bounded overspending (BOS) (Papasotiropoulos et al., 2025).

Our analysis indicates that certain variants of our rules perform particularly well across all metrics. Under the cost-utilities approach, both MTS and MTC consistently achieve strong results: MTC tends to select smaller projects than MTS and slightly improves the exclusion ratio, though at the expense of lower total cost-utility. Notably, neither rule ever violates EJR+ up to one, and in many respects they are comparable to Equal Shares and BOS. Compared with these two methods, MTS selects larger projects and attains higher cost-utility in medium and large elections, while in smaller elections it lies between Equal Shares and BOS. Among the elimination-with-transfers rules, EwTC provides the best balance between proportionality and total cost-utility. Under the score-utilities approach, MTS consistently outperforms MTC in terms of total cost-utility. Overall, we believe that MTS exhibit particularly good properties.

## Related work

There is a large body of work on participatory budgeting (PB) and related topics in the social choice literature (see, e.g., Aziz and Shah (2020); Rey and Maly (2023)), which relies on different assumptions. A first point of divergence is the type of information solicited from voters. Since most PB aggregation rules adapt existing voting rules, the elicited input usually follows standard social choice formats: voters are asked either to approve projects (and thus disapprove others) or to rank projects by desirability. In approval ballots, voters are typically asked either to approve a fixed number of projects (say *k*) (Faliszewski & Talmon, 2019) or any subset of projects whose total cost does not exceed the budget (knapsack voting) (Goel et al., 2019). Goel et al. (2019) also considered pairwise comparisons, asking voters to compare projects by their value-to-cost ratio. These comparisons are then aggregated using variants of classic voting rules such as Borda and Kemeny. Klamler et al. (2012) and Lu and Boutilier (2011) proposed adaptations of multiwinner voting rules that, given project rankings, select *k* alternatives subject to their costs and the budget constraint. In these models, rankings are independent of costs. A number of works have instead solicited cardinal utilities for projects (Fain et al., 2018; Fluschnik et al., 2019; Peters et al., 2021; Papasotiropoulos et al., 2025), with voters specifying their utility for the implementation of each project. Benade et al. (2021) introduced a knapsack-style model in which voters support the bundle maximizing their utility. They also suggested other elicitation formats: Value voting (ranking by value), Value-for-money voting (ranking by value-to-cost), and Threshold voting (supporting all projects exceeding a given value threshold). Laruelle (2021) proposed that such utilities may be implicit and inferred from rankings. The question of proportional representation in PB naturally leads to fairness considerations. For instance, it should not happen that no project supported by a large minority is funded. The notion of justified representation for approval-based PB rules was introduced in Aziz and Lee (2018); Peters et al. (2021); Papasotiropoulos et al. (2025), while proportionality for solid coalitions in the ordinal setting was studied in Aziz and Lee (2019). Cumulative and cardinal ballots have also been explored in divisible models of PB, where voters allocate fractions of the budget across projects rather than selecting projects outright (Fain et al., 2016; Freeman et al., 2019). A broader literature on cumulative voting exists, but it mostly concerns single-winner elections and focuses on practical or legal aspects (Mills, 1968; Bhagat & Brickley, 1984; Cole Jr, 1949; Vengroff, 2003). There is also extensive research on the Single Transferable Vote (STV). It is widely regarded as a strong rule for both single-winner and multiwinner elections, especially when proportional representation is desired; see, e.g., Tideman and Richardson (2000); Elkind et al. (2017, 2017). This motivated us to adapt STV to PB via cumulative voting. Relatedly, Ford [Ford (2020), Section 3.4] proposed a cumulative version of STV for multiwinner elections. The work most closely related to ours is the *Accurate Democracy* project.^6^ It describes an iterative PB procedure reminiscent of STV. However, the description provided is informal, and the proposed rule differs from ours in several respects–for example, it combines ordinal and cumulative ballots. Moreover, no axiomatic analysis is offered.

## Conclusion

We proposed the use of cumulative ballots for participatory budgeting and analyzed several aggregation methods within this framework. Our results–particularly the experimental findings presented in Tables 2, 3, 4–indicate that MTS and MTC deserve serious consideration in practical applications, for the following reasons:

- Both MTS and MTC satisfy Strong-PR and are therefore guaranteed to achieve highly proportional outcomes. In particular, they ensure minority representation, in contrast to the methods commonly used in practice.
- Both rules are computationally efficient: their procedural descriptions are simple, and this efficiency is confirmed by our simulations.

Looking forward, we see several promising directions for extending CSTV to more general PB settings. While in this paper we focused on the standard combinatorial PB model, cumulative ballots and the CSTV framework can be naturally adapted to richer ballot structures, enabling greater voter expressiveness and potentially yielding even better outcomes. In particular, we plan to investigate the following extensions:

**PB with structured projects.** Some projects may depend on others being implemented first. Such dependencies, representable as a graph known to the voters, could be integrated into the CSTV framework.

**PB with project interactions.** Projects may interact, e.g., as in the model of Jain et al. (2020), where a *substitution structure* partitions the projects. Beyond cumulative ballots, voters could express preferences over substitutions and complementarities. Since CSTV redistributes support on behalf of voters, it could be extended to let voters explicitly direct how such redistribution should occur.

**PB with multiple resource types.** In some settings, resources are multidimensional (e.g., time and money). Voters could then be given several types of “virtual coins” to allocate simultaneously.

**Negative utilities.** Voters may wish not only to support but also to oppose certain projects. Here, cumulative ballots could allow voters to split coins between positive and negative support. Finally, our work highlights interesting open questions. In particular, does there exist a proportional rule that satisfies support monotonicity, or are the axioms we study inherently incompatible? More broadly, we believe that weaker notions of monotonicity and resilience against project splitting and merging deserve careful investigation.

## Data Availability

All data utilized in this study is publicly available and can be found in the Pabulib library: https://pabulib.org.

## Appendix

### Theorem 1

GS, EwTC, EwT, EwTS, MTS and MTC do not satisfy splitting monotonicity, while GSC, and MT satisfy the property.

### Proof

**GS**: Consider the following budgeting instance with one voter and 2 projects, $p_1$ and $p_2$. Assume that $c(p_1) = c(p_2) = 2$ and that the budget is $L = 2$. The cumulative ballot of the single voter *v* is $v(p_1) = 0.6$ and $v(p_2) = 0.4$. GS would select $p_1$. Now, assume that we split $p_1$ into two projects, $p_a$ and $p_b$, such that $c(p_a) = c(p_b) = 1$ and such that $v(p_a) = v(p_b) = 0.3$. Now, GS would select $p_2$, failing splitting monotonicity.

**GSC**: First, observe that in order to prove that a rule satisfies splitting monotonicity it is sufficient to consider cases where the project that is to be split is divided into two parts. Indeed, if the project were divided into more than two parts, one could use the reasoning for splitting into two projects recursively.

Consider a project *p* that is about to be split into $p_a$ and $p_b$. Observe that one of the two following inequalities must hold

$$\begin{aligned} \frac{\textrm{support}(p_a)}{c(p_a)} \ge \frac{\textrm{support}(p)}{c(p)}, \quad \quad \frac{\textrm{support}(p_b)}{c(p_b)} \ge \frac{\textrm{support}(p)}{c(p)}. \end{aligned}$$

Indeed, if none of the above inequalities held, then we would have:

$$\begin{aligned} 1 = \frac{\textrm{support}(p_a) + \textrm{support}(p_b)}{\textrm{support}(p)} < \frac{c(p_a)}{c(p)} + \frac{c(p_b)}{c(p)} = 1 \text { ,} \end{aligned}$$

a contradiction. W.l.o.g., let us assume that:

$$\begin{aligned} \frac{\textrm{support}(p_a)}{c(p_a)} \ge \frac{\textrm{support}(p)}{c(p)}\ \text {.} \end{aligned}$$

Assume that *p* is selected by the rule. Then, after the split, $p_a$ would be selected at the same moment as *p* or before. This proves that GSC satisfies splitting monotonicity.

**EwT, EwTC, EwTS**: Consider the following instance with 3 projects, $p_1, p_2$, and $p_3$. Their costs are $c(p_1) = 17$, $c(p_2) = 12$, and $c(p_3) = 20$, and the budget is $L = 20$. Let us fix a small constant $\epsilon$. There are 20 voters with the cumulative ballots given in the following table:

**Table:**

# votes	$p_1$	$p_2$	$p_3$
15	$\nicefrac {8}{15}$	$\nicefrac {7}{15}$	0
5	$\nicefrac {1}{5} - \epsilon$	$\epsilon$	$\nicefrac {4}{5}$

The support of $p_1, p_2$, and $p_3$ equals respectively, $9 - 5\epsilon$, $7 + 5\epsilon$, and 4. Thus, $p_3$ will be eliminated first. The last 5 voters will transfer (almost) the entire support to $p_1$. Thus, $p_2$ will be eliminated next, and so $p_1$ will be selected.

Now, assume that $p_1$ is split into $p_a$ and $p_b$ such that $c(p_a) = 8.5$, and $c(p_b) = 8.5$. The voters’ preferences would be as follows:

**Table:**

# votes	$p_a$	$p_b$	$p_2$	$p_3$
15	0	$\nicefrac {8}{15}$	$\nicefrac {7}{15}$	0
5	$\nicefrac {1}{5} - \epsilon$	0	$\epsilon$	$\nicefrac {4}{5}$

Then, $p_a$ is eliminated first, and the last 5 voters transfer their almost entire support from $p_a$ to $p_3$. Next, $p_3$ is eliminated, and the whole value of the last 5 voters is transferred to $p_2$. Thus, the total support of $p_2$ becomes 12. In the last step $p_2$ is selected, and neither $p_a$ nor $p_b$ fit within the remaining budget. The splitting monotonicity does not hold.

**MT**: First, we observe that the sum of transfers to each project stays the same after the split. If at some point *p* was eligible for funding, then at this point $p_a$ or $p_b$ would be as well, and so one of these projects would be selected. Thus, from now on, let us assume that the excess of *p* was always negative. Observe that if $p_a$ or $p_b$ becomes noneligible by transfers, then this means that the other project’s excess is greater than the excess of *p*. Thus, there always exists a project, among the two, with the excess value greater or equal to the excess value of *p* that is eligible by transfers. Thus, $p_a$ or $p_b$ will be selected at most at the time when *p* was.

**MTC, MTS**: Consider the instance with 2 projects, $p_1$ and $p_2$, with costs equal to $c(p_1) = c(p_2) = 150$. The budget is $L = 200$. There are 200 voters with the following preferences:

**Table:**

# votes	$p_1$	$p_2$
150	$\nicefrac {1}{3} + \epsilon$	$\nicefrac {2}{3} - \epsilon$
50	1	0

In this example, the two projects are both eligible by transfers, thus MTC and MTS would select the one with the higher support, i.e., $p_1$.

Now, assume that $p_1$ is split into $p_a$ and $p_b$ such that $c(p_a) = 51$, and $c(p_b) = 99$. The voters’ preferences look as follows:

**Table:**

# votes	$p_a$	$p_b$	$p_2$
150	0	$\nicefrac {1}{3} + \epsilon$	$\nicefrac {2}{3} - \epsilon$
50	1	0	0

After the split, $p_a$ is not eligible by transfers; $p_2$ will be selected, leaving no room for $p_a$ nor $p_b$. $\square$

The next axiom is analogous to the previous one, yet it is about merges among the projects rather than splits.

### Theorem 2

GS satisfies merging monotonicity, while EwT, EwTS, EwTC, MT, MTS, MTC, GSC fail merging monotonicity.

### Proof

**MT, MTS, MTC**: Consider an instance with 5 projects, *p*, *q*, *r*, *s*, and *z* with all the costs equal to 10. The first 10 voters put the value $0.5 + \epsilon$ to *p* and $0.5 - \epsilon$ to *q*. The next 9 voters put $0.5 + \epsilon$ to *r* and $0.5 - \epsilon$ to *s*. The last voter puts 1 to *z*. There are $n = 10 + 9 + 1 = 20$ voters; the budget is $L = 20$.

Since *p* and *q* are eligible by transfers, MT, MTS and MTC will select *p* first. For that, most of *q*’s money will be transferred to *p* and as a result all rules will select *r* in the second iteration. Now, assume that *p* and *r* are merged into *x*. The merged project is no longer eligible by transfers, but *q* is still. It will be selected, and there will be no money left in the budget to buy *x*.

**EwT, EwTS, EwTC**: Consider the following instance with 5 projects, *p*, *q*, *r*, *s*, and *z*. The costs of all the projects are equal to 30. The first 30 voters assign value 1 to *p*. The next voter assigns value $1 -\epsilon$ to *p* and $\epsilon$ to *q*. The next 15 voters assign $1-2\epsilon$ to *q* and $2\epsilon$ to *r*; the next 30 voters assign $0.5 - \epsilon$ to *r* and $0.5 + \epsilon$ to *s*; the remaining 14 voters assign 1 to *z*. The number of voters is 90 and the budget is $L = 90$. The cumulative ballots of the voters are summarized in the table below.

**Table:**

# votes	*p*	*q*	*r*	*s*	*z*
30	1	0	0	0	0
1	$1-\epsilon$	$\epsilon$	0	0	0
15	0	$1-2\epsilon$	$2\epsilon$	0	0
30	0	0	$0.5 - \epsilon$	$0.5 + \epsilon$	0
1	0	0.33	0.33	0.33	0
13	0	0	0	0	1

Here, EwT, EwTS and EwTC will select *p* first. The value of the 31st voter will be transferred to *q*. Then, the support for *q*, *r*, and *s* will be $16.33 - 30\epsilon$, 15.33, and $15.33+ 30\epsilon$, respectively. Thus, *z* and *r* will be eliminated next; there will be enough money to accommodate the remaining two projects, thus *p*, *q* and *s* will be selected.

Now assume that *p* and *s* are merged into *x*: it does not reach the threshold. Indeed, the support of projects *x*, *q* and *r* will be $46.33 +29\epsilon$, $15.33 - 29\epsilon$, and 15.33, respectively. Thus, *z* and *q* will be eliminated next. The total value of $15 - 30\epsilon$ will be transferred from *q* to *r*, raising its support to $30.33 - 30\epsilon$. Thus, *r* will be chosen, leaving no money in the budget for *x*.

**GSC**: Consider an instance with 3 projects $p_1$, $p_2$, and $p_3$ with the costs equal to, respectively, 5, 10, and 5. The budget is $L = 10$. There are 10 identical voters, who assign value 0.35 to $p_1$, 0.6 to $p_2$ and 0.05 to $p_3$. GSC selects $p_1$ first, and then there will be no money for $p_2$. Consequently, $p_1$ and $p_3$ will be selected. On the other hand, if we merge $p_1$ and $p_3$, then the rule will select $p_2$.

**GS**: Observe that merging two projects does not affect the order of consideration of the other projects apart from the merged ones. Assume we merged $p_a$ and $p_b$ into *p* and that $p_b$ was considered after $p_a$ by the rule. Thus, *p* will be considered in the same or earlier iteration as $p_a$, and there will be enough money to accommodate *p* (since at this moment, before merge, there was enough money to add $p_a$ and $p_b$). $\square$

### Theorem 3

GS, GSC, EwT, EwTS, EwTC, MT, MTS, and MTC fail support monotonicity.

### Proof

**EwT, EwTS, EwTC**: Consider the instance with 3 projects, *p*, *q*, *r*. The costs of all projects are equal to 10. The first 8 voters put $0.5 - \epsilon$ to *p*, 0.25 to *q* and $0.25 + \epsilon$ to *r*. The next 2 voters put 1 to *q* and the next 2 to put 1 to *r*. There are 12 voters and the budget is $L = 12$.

Here, *p* is eliminated first, *q* is eliminated second, and so *r* is selected for funding.

Now, assume that all the voters move $2\epsilon$ from *q* to *r*. Now, *q* is eliminated first and its value is redistributed among *p* and *r* in proportions 2 : 1. Thus, *r* is eliminated second, and so *p* is the project selected for funding.

**MT**: Assume we have 3 projects, *p*, *q* and *r*, with the costs equal to 4, 4 and 8, respectively. There are 10 identical voters who put utility $0.25+\epsilon$ on *p*, 0.1 on *q*, and $0.65-\epsilon$ on *r*. The budget is $L = 10$.

MT will select *p* first. Since there will be no money left for *r*, *q* will be selected as a second project for funding. Now, assume that each voter moves $2\epsilon$ from *p* to *q*. Now, MT selects *r* first and there will be no money left for *q*.

**GS, MTS**: The instance is similar as for MT. The projects have the same costs but the voters put $\nicefrac {1}{3}+2\epsilon$ on *p*, $\nicefrac {1}{3} + \epsilon$ on *r* and $\nicefrac {1}{3}-3\epsilon$ on *q*. Here GS and MTS will select *p* and *q*. Now assume that the voters $2\epsilon$ from *p* to *q*. After such a change GS and MTS will select *r*.

**MTC, GSC**: The constructions here are analogous to MT and GS. $\square$

### Theorem 4

GSC satisfies Weak-PR but GS fails it.

### Proof

**GS**: Consider a scenario with $P = \{a, b\}$, $c(a) = 1$, $c(b) = 3$, $L = 3$, and voters $v_1$, $v_2$, and $v_3$, where voter $v_1$ supports only *a*, and $v_2$ and $v_3$ support only project *b*. For $\ell = 1$, voter $v_1$ acts as a set $V'$ of $|V'| \ge \ell n / L = 1$ and $P' = \{a\}$ acts as a set of projects with $c(P') \le \ell$; GS, however, selects only project *b*, as project *b* has higher support and, after *b* is funded, no funds are left to fund project *a*.

**GSC**: Let $\ell \in [L]$. Consider a set $V'$ of voters with $|V'| \ge \ell n / L$ and a set $P' \subseteq P$ of projects with $c(P') \le \ell$. Set the vote of each $v' \in V'$ to support only the projects in $P'$, proportionally: i.e., for each $p' \in P'$ set $v'(p') = c(p') / c(P')$ and $v'(p) = 0$ for each $p \notin P'$. Now, the sum of support each project $p' \in P'$ gets from the voters is at least $|V'| \cdot c(p') / c(P')$, as it gets this amount already from the voters in $V'$. As $|V'| \ge \ell n / L$, we have that the sum of support of each project $p' \in P'$ is at least $\ell n / L \cdot c(p') / c(P')$; furthermore, since $c(P') \le \ell$, it follows that this sum of support is at least $n / L \cdot c(p')$.

GSC ranks projects according to their sum of support over their cost, so the “support over cost” value of each $p' \in P'$ is at least *n*/*L*. We wish to upper bound the total cost of projects $p \notin P'$ which get a “support over cost” value greater than *n*/*L*: The proof will follow by showing that the total cost of such projects is at most $L - \ell$, because then, GSC would fund all projects $p' \in P'$. To show this, assume otherwise, that the total cost of projects $p \notin P'$ with “support over cost” value greater than *n*/*L* is more than $L - \ell$, call the set of these projects *S*.

Observe that the number of voters $v \notin V'$ is at most $n - \ell n / L$. Thus, the total support divided by the total cost must be lower than $\frac{n - \frac{\ell n}{L}}{L - \ell } = \frac{n}{L}$; hence, contradiction. $\square$

### Theorem 5

EwT, EwTS, and EwTC satisfy PR but GSC fails it.

### Proof

**GSC**: Intuitively, GSC fails PR because after one project in $|P'|$ is funded, its excess gets lost, which might cause the other projects in $P'$ not funded.

More formally, consider a budgeting scenario with $P = \{a, b, c\}$ where $c(a) = c(b) = 1$ and $c(c) = 3$, the budget limit $L = 4$, and voters $v_1$ and $v_2$, where voter $v_1$ assigns to project *a* value $1 - \epsilon$ and to project *b* value $\epsilon$, and voter $v_2$ assigns to project *c* value 1.

According to PR, with $V' = \{v_1\}$, $\ell = 2$, and $P' = \{a, b\}$, we have that indeed both *a* and *b* shall be funded. GSC, however, will choose the bundle $\{a, c\}$.

**EwT, EwTS, EwTC**: Let $P' \subseteq P$ be a set of projects, and let $V'$ be a group of voters who all support all projects from $P'$ and no other projects; assume $c(P') \le L \cdot \nicefrac {|V'|}{n}$.

Recall that we define the support of a project *p* as:

$$\begin{aligned} \textrm{support}(p) = L \cdot \frac{\sum _{j=1}^n v_j(p)}{n} \text { . } \end{aligned}$$

Let $S_i$ be the set of projects picked by the rule (EwT or EwTC) up to the *i*th iteration, inclusive. We prove the following invariant: In the *i*th step the total support the voters from $V'$ assign to the projects from $P' \setminus S_i$ equals at least $|V'| \cdot \nicefrac {L}{n} - c(P' \cap S_i)$ and no project from $P'$ has been eliminated by the rule. The invariant is clearly true when the rule begins. Now, assume it is true after the *i*th iteration, and we will show that it must hold after the $(i+1)$th iteration as well. Observe that in the $(i+1)$th iteration the total support of the candidates from $P'$ equals at least:

$$\begin{aligned} |V'| \cdot \nicefrac {L}{n} - c(P' \cap S_i) \ge c(P') - c(P' \cap S_i) = c(P' \setminus S_i) \text { .} \end{aligned}$$

Thus, there must exists at least one project, support of which exceeds the cost, thus no project from $P'$ can be eliminated. Furthermore, if a project $p' \in P'$ is selected, then the amount of support that the voters from $V'$ assign to the projects from $P'$ will decrease by $c(p')$ (the exceed will be transferred only to the projects from $P'$, unless all of them are already selected). This proves the invariant. Since no project from $P'$ will be eliminated, all of them will be picked by the rule. $\square$

### Theorem 6

MT, MTS, and MTC satisfy Strong-PR but EwT, EwTS and EwTC fail it.

### Proof

**EwT, EwTS, EwTC**: Consider an instance with 10 voters, and 3 projects, $p_1, p_2$, and *q*, such that $c(p_1) = 4$, $c(p_2) = 7$, and $c(q) = 7$. The budget limit is $L = 10$. The first four voters put $\epsilon$ to $p_1$ and $1-\epsilon$ to $p_2$. The next six voters put the value of 1 to *q*. The support of the projects $p_1, p_2$, and *q* will be respectively, $4\epsilon$, $4 - 4\epsilon$, and 6. Thus, EwT, EwTS, and EwTC will eliminate $p_1$ first, $p_2$ second, and *q* last. Consequently, only *q* will be selected while Strong-PJR requires selecting $p_1$.

**MT, MTS, MTC**: Consider a group of voters $V'$, and a set of projects $P' \subseteq P$, which are supported by all the voters from $V'$; furthermore, assume the voters from $V'$ do not support any other projects.

Let $S_i$ denote the set of projects selected by the rule (MT, MTS, or MTC) up to the *i*th iteration, inclusive. First, we observe that the following invariant holds: In each iteration *i* the total support that the voters from $V'$ assign to the projects from $P' \setminus S_i$ equals at least $L \cdot \nicefrac {|V'|}{n} - c(S_i \cap P')$. Indeed, the invariant is initially true (since $c(S_0 \cap P') = 0$), and each time a project $p \in P'$ is selected, the total support that voters from $V'$ assign to projects is decreased by at most *c*(*p*). Furthermore, the excess of the value that the voters from $V'$ assign to *p* is redistributed only among the projects from $P'$.

Now, for the sake of contradiction, assume there exists a project $p' \in P'$ such that $c(p') + c(P' \cap \mathcal {R}(E)) \le L \cdot \nicefrac {|V'|}{n}$ and that has not been selected. Let *j* be the last iteration before the rule reached the “Inclusive Maximality Postprocedure” phase (possibly *j* is the last iteration of the rule). Then, clearly $c(p') + c(P' \cap S_j) \le L \cdot \nicefrac {|V'|}{n}$. By our invariant, we get that in the *j*th iteration the total support the voters from $V'$ assign to the projects from $P' \setminus S_j$ equals at least:

$$\begin{aligned} L \cdot \nicefrac {|V'|}{n} - c(S_j \cap P') \ge c(p') \text { .} \end{aligned}$$

Furthermore, all the voters from $V'$ support $p'$, thus $p'$ is eligible by transfers. Consequently, the rule cannot stop nor reach the “Inclusive Maximality Postprocedure” phase. This gives a contradiction and completes the proof. $\square$

## Footnotes

1 The websites http://www.participatorybudgeting.org and http://pbstanford.org provide up-to-date information on the adoption of participatory budgeting in North America. A broader list of cities implementing PB worldwide can be found at http://en.wikipedia.org/wiki/Participatory_budgeting_by_country.

2 A ranking of the projects can be transformed into a cumulative ballot by applying a positional scoring function, such as Borda, to the ranking and normalizing the resulting scores.

3 An alternative approach would be to interpret $v_j(p)$ as the fraction of the funds available to voter *j* that she thinks should be assigned to project *p*. These two interpretations are close and differ in whether we take the local or the global view on the voters’ preferences. In this paper we take the local interpretation—we assume that the voters indirectly control the funds, and indicate how parts of funds that they control should be spread among the projects.

4 If *f* is not injective, ties are resolved using a fixed, arbitrary tie-breaking rule.

5 The greedy rule can be interpreted as a greedy algorithm tailored to optimize this metric.

6 https://www.accuratedemocracy.com/p_need2.htm
